# Structural Design of MEMS Acceleration Sensor Based on PZT Plate Capacitance Detection

**DOI:** 10.3390/mi14081565

**Published:** 2023-08-06

**Authors:** Min Cui, Senhui Chuai, Yong Huang, Yang Liu, Jian Li

**Affiliations:** 1 Shanxi Key Laboratory of Information Detection and Processing, North University of China, Taiyuan 030051, China; 2 School of Instrumentation and Electronics, North University of China, Taiyuan 030051, China; sz202206204@st.nuc.edu.cn (S.C.); liuyang03042022@163.com (Y.L.); 3 Shanghai Institute of Aerospace Control Technology, Shanghai 201109, China; huangyong9608@163.com; 4 School of Information and Communication Engineering, North University of China, Taiyuan 030051, China; lijian@nuc.edu.cn

**Keywords:** PZT, layer counting identification, capacitive acceleration sensor, MEMS, LS-DYNA

## Abstract

The problem that the fuze overload signal sticks and is not easily identified by the counting layer during the high-speed intrusion of the projectile is an important factor affecting the explosion of the projectile in the specified layer. A three-pole plate dual-capacitance acceleration sensor based on the capacitive sensor principle is constructed in this paper. The modal simulation of the sensor structure is carried out using COMSOL 6.1 simulation software, the structural parameters of the sensor are derived from the mechanical properties of the model, and finally the physical sensor is processed and fabricated using the derived structural parameters. The mechanical impact characteristics of the model under different overloads were investigated using ANSYS/LS-DYNA, and the numerical simulation of the projectile intrusion into the three-layer concrete slab was carried out using LS-DYNA. Under different overload conditions, the sensor was tested using the Machette’s hammer test and the output signal of the sensor was obtained. The output signal was analyzed. Finally, a sensor with self-powered output, high output voltage amplitude, and low spurious interference was obtained. The results show that the ceramic capacitive sensor has a reasonable structure, can reliably receive vibration signals, and has certain engineering applications in the intrusion meter layer.

## 1. Introduction

In modern war, in order to prevent important targets from being destroyed by the enemy, targets of strategic significance (underground command center, missile silo, weapons depot, underground communication hub, etc.) are often hidden underground with extremely strong fortifications [1]. For example, the Japanese Central Command Post, which was completed and put into use in 1984, has two floors above ground and three underground, with a total depth of more than 30 m [2]. The Command and Operation Center of the French Staff Headquarters is a two-story and half-story building composed of steel bars and high-strength concrete, with the lowest ground level at −10 m [3]. The underground command center of the Iraqi Presidential Palace is 18 m above the ground, and its main structure is made of special high-strength and heat-resistant concrete with a thickness of up to two meters [4]. Therefore, how to effectively attack this kind of target has become the focus for various countries and military departments [5]. Various research institutions are constant committed to finding more effective means and methods of damage [6]. Based on this goal, penetration ammunition was born. Penetration ammunition mainly relies on the kinetic energy of the warhead to invade the inside of the target; after penetrating the target, the intelligent fuze to controls the location of the projectile blast point to achieve the maximum damage effect [7]. Penetration munitions can be used in various weapon systems [8], including air force weapons such as aerial bombs and air-to-ground missiles, lethal weapons such as strategic guided missiles and ground-to-ground missiles, and naval weapons such as ship-borne cruise missiles, ship-to-ground missiles, and so on [9]. In general, penetration munitions are aimed at high-value, hardened targets and are therefore mostly used in a variety of precision strike weapons [10]. According to the types of targets, penetration munitions can be used to target important ground targets such as airport runways, bridges, ground command centers, and communication hubs. They can also effectively strike various underground command centers, missile silos, weapons depots, and underground communication hubs [11]. They can also strike a variety of maritime targets, including the precision killing of aircraft carrier fleets, frigates, and reef military bunkers [12].

Due to the in-depth research on high-speed and ultrahigh-speed intrusion munitions technology at home and abroad, the multi-layer target intrusion layer counting problem of high-speed large aspect ratio projectiles has become a hot topic of research in the field of intrusion at home and abroad [13]. The MEMS island-beam acceleration sensor has the technical advantage of a high range [14], and its layer counting sensing technology is usually applied to the multi-layer target layer counting of high-speed and large aspect ratio projectiles (initial velocity of intrusion greater than 600 m/s). However, the following problems still exist:

When the aspect ratio of the projectile increases and the projectile velocity is greater than 800 m/s, the stress wave propagates back and forth in the projectile body during the penetration process, resulting in concussive acceleration [15]. The peak value of the high-frequency concussive signal will increase rapidly and the overload envelope of multi-layer target penetration will be completely submerged, resulting in the mutual adhesion of overload signals from layer to layer, which cannot effectively identify the penetration layer [16].

The problem of signal adhesion is difficult to solve at the root. The maximum destructive effect of an intrusive munition must be achieved by intelligent fuze control and detonation at the right location [17]. A fuze is a special single-use product defined as a control system that can use information about the target, environment, platform, and network to detonate or ignite the combatant charge according to a predetermined strategy. It can select the point of detonation and give instructions for range or extended engine ignition, and information about the destructive effect [18]. There are four commonly used fuze initiation methods for intrusion munitions: timed initiation, layer/cavity initiation, stroke initiation, and media recognition initiation [19]. Among the four initiation methods, the layer/cavity counting initiation is the most widely studied and the most intensively researched initiation method. The principle of layer/cavity counting identification fuze is that when the strike target is a multi-layer hard target, the acceleration sensor in the fuze receives the acceleration signal output penetrated each hard target layer, so that the electrical signal system inside the fuze processes the acceleration signal (the size of the deceleration, derivative, inflection point, etc.); and analyze the number of layers of the target that have penetrated the target to drive the position of the projectile. When the projectile reaches a predetermined number of target layers, the fuze emits a detonation signal and detonates the explosive to achieve the optimal destruction of the penetrated target [20]. The existing fuze layer counting techniques often rely on single-axis acceleration sensors to sense the overload of the projectile as it penetrates each layer of the target and the calculation of the number of target layers is achieved by using a comparison of the overload signal amplitude and threshold; great progress has been made in engineering applications. However, the complexity of the penetration problem, including the oscillation of the projectile, the friction between the projectile and the target plate, and the characteristics of the sensor, can lead to a large amount of clutter interference in the overload signal, and in some cases the overload signal amplitude of the penetrated target is not obvious, leading to difficulties in fuze layer counting or even layer counting errors Therefore, in order to further improve the accuracy of the fuze layer, it is necessary to explore better sensors to solve the interference problem at the source of the overload signal generation and to study more accurate and reliable fuze layer counting strategies. In this paper, we focus on the precise identification of multilayer targets for target penetration munition fuzes. In the layer counting identification of an intruder fuze, the main layer counting method currently relies on high g-value acceleration sensors or an acceleration threshold switch to realize layer counting; however, there are two very important key points to accurately count the number of layers through which the intruder warhead has passed:

(1) The occurrence of the overload signal
(2) The way the overload signal is processed and discriminated.

This paper focuses on the precise identification of multilayer targets for intrusion munitions fuzes. A flat plate capacitive acceleration sensor based on a three-layer pole plate is designed for the layer counting recognition of intrusion munition fuzes. The corresponding simulation and tests are conducted to obtain a sensor with excellent output performance.

## 2. Design Principle

### 2.1. Three-Dimensional Acceleration Numerical Simulation of Projectile Penetration into a Multilayer Concrete Target

With the continuous development of modern science, especially the development of computer technology and various intrusion theories, numerical simulation by computers has become a very critical and important technology [21]. The penetration problem is a very complex problem, involving the calculation of the deformation of the bullet and the target plate, material stress, target plate erosion, and a series of key points. The previous empirical and analytical methods have certain shortcomings [22]. The empirical method requires a large number of live firing tests and analysis of the measured data, which requires huge manpower and material resources and is less economical [23]. The analytical method requires the establishment of certain prerequisites, and the scope of application is more restricted [24]. The numerical method relies on electronic computers and combines the concept of finite elements or finite volumes to achieve the purpose of studying engineering and physical problems and even various problems in nature by means of numerical calculations and image display [25].

In order to accurately verify the rationality and usability of the designed sensor, LS-DYNA 19.2 finite element simulation software was used to produce and solve the whole process of projectile intrusion into the concrete target plate and obtain the overload signal of the smart fuze inside the projectile during the projectile intrusion, which supports the accurate layer counting identification of the subsequent fuze [26].

Firstly, the LS-DYNA finite element simulation software was used to simulate the dynamics of the projectile body penetrating the three-layered cement board.

**Figure 1:**
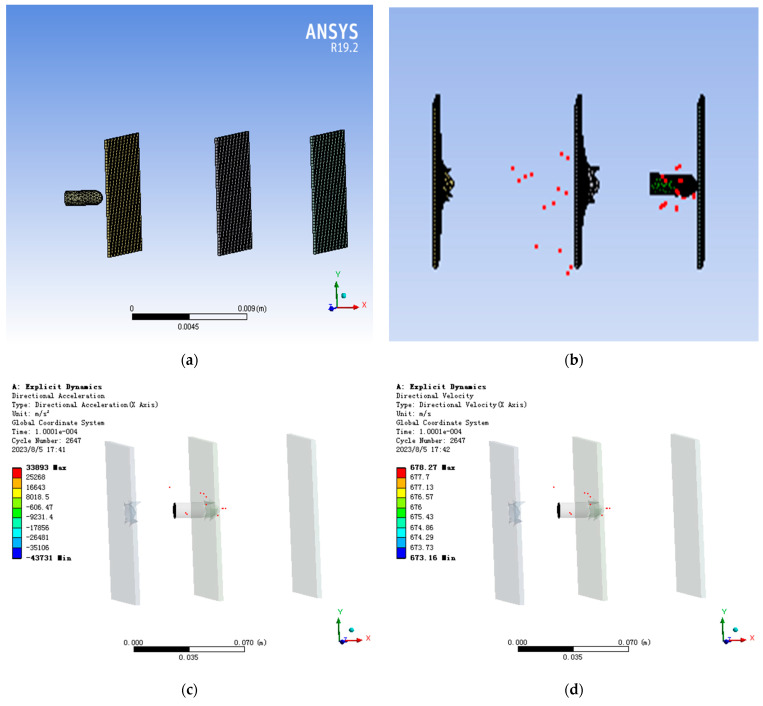
Simulation diagram of projectile penetration of three-layer plate: (**a**) is the physical model of the projectile penetrating the three-layer target, (**b**) is the penetration diagram when the acceleration is whole, (**c**) is the penetration diagram of the tail velocity, and (**d**) is the penetration diagram of the tail acceleration.

When designing the projectile penetration model, the initial velocity of the projectile was set to 800 m/s, the thickness of the reinforced concrete target plate was 40 mm, the spacing of the target plate was 300 mm, 400 mm, or 500 mm, the diameter of the projectile body was 60 mm, and the vertical length was 200 mm; the penetration model is shown in Figure 1.

**Figure 2:**
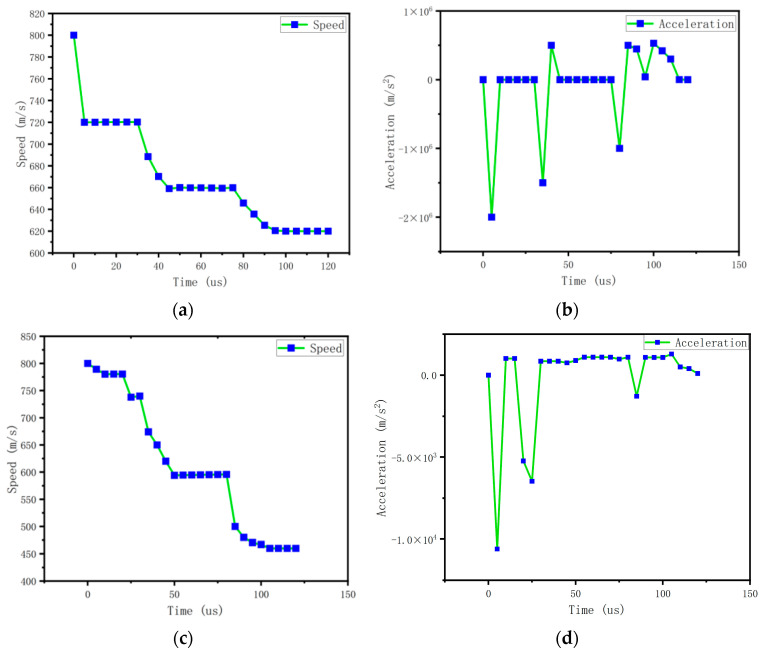
The overall velocity and acceleration curves of the projectile, as well as the velocity and acceleration curves of the projectile: (**a**) is the velocity of the projectile as a whole, (**b**) is the acceleration of the projectile as a whole, (**c**) is the velocity of the projectile tail, and (**d**) is the acceleration of the projectile tail.

The velocity and acceleration changes of the projectile penetration simulation are shown in Figure 2. Figure 2a,b show the changes in projectile velocity and acceleration during the penetration process. According to the images, when the projectile completely penetrates the first target plate, the horizontal velocity decreases from 800 m/s to 724.39 m/s; the velocity difference is 75.61 m/s and the maximum acceleration is −257,176.87 g. When the projectile penetrates the second target plate, the horizontal velocity decreases to 659.3 m/s, the velocity difference is about 65.09 m/s, and the maximum acceleration is −189,766.764 g. When the projectile completely penetrates the third target plate, the horizontal velocity decreases to 619.31 m/s, the velocity difference is about 39.99 m/s, and the maximum acceleration is −116,588.921 g.

We place the probe at the rear of the projectile to detect the velocity and acceleration of the projectile during penetration. The probe diagram is shown in Figure 1c,d. Figure 2c,d show the velocity and acceleration curves at the rear of the projectile. From Figure 2d, it can be seen that when the projectile penetrates the cement plate, due to the internal structure of the projectile, the maximum acceleration caused by the penetration resistance propagating to the rear of the projectile is about 10,000 g.

The core of this layer counting principle approach lies in the extraction of acceleration peaks and the setting of the thresholds. Using the intrusion into multiple layers of targets, the acceleration will suddenly change to produce a peak and the counting of the number of layers penetrating the target is achieved by comparing the magnitude of this peak with a set threshold [27].

It can be seen that the projectile took about 120 µs from the beginning of contacting the first target plate to leaving the last target plate and that the projectile decreased by a total of 180 m/s during the process of penetrating the three target plates. Overall, the acceleration overload values of the three layers of penetration were large and the acceleration sensors could easily obtain their corresponding acceleration signals.

### 2.2. Principle of Capacitance Sensor Measurement

The sensor element of a capacitive sensor is various types of capacitors. When the external measurement changes, it leads to the change in capacitor capacitance. Through the corresponding external measurement circuit, the capacitance change of a capacitor is converted into an electrical signal through the corresponding mathematical relationship. Then, by measuring the size of electrical signals, the changes and sizes measured can be judged [28].

**Figure 3:**
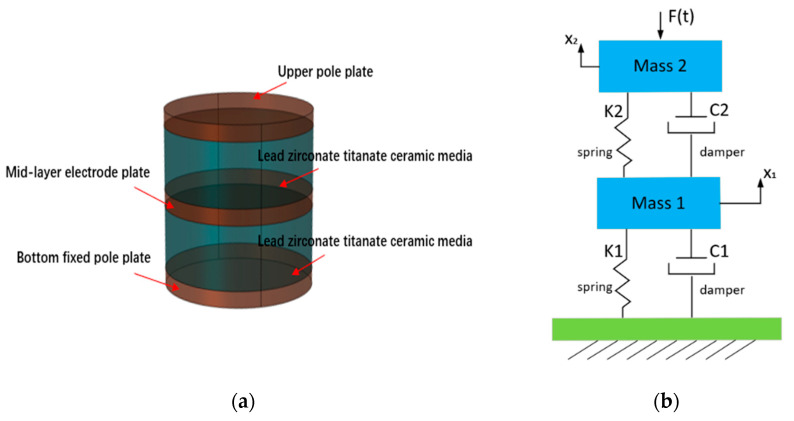
Overall structure of double-plate capacitive accelerometer and equivalent dynamics model of the double-plate capacitive accelerometer. (**a**) Overall structure of the three-electrode plate capacitance accelerometer and (**b**) equivalent kinetic model.

The capacitive acceleration sensor designed in this paper has a double-capacitance structure. The overall structure is cylindrical, consisting of movable upper and middle plates, a fixed lower plate, and upper and lower zirconia ceramic dielectric layers, the diameter of the plates and dielectric layers is 4.5 mm, the thickness of the three plates is 0.5 mm, and the thickness of the ceramic dielectric layer is 1.5 mm. Its structure is shown in Figure 3a, and the equivalent dynamics model is shown in Figure 3b.

The traditional acceleration sensor often adopts a single cantilever beam, a double cantilever beam, or piezoresistive structure [29]. The sensor internal sensitive element also has the property of material damping after deformation, but due to its own structural reasons, the material damping area is small, resulting in the shock attenuation of the sensor after the impact; if the shock signal lasts a long time, the sensor cannot quickly restore to the initial state [30]. Based on the above reasons, the structure of the sensor was changed, the damping area of the material was increased, and the damping of the sensor was increased so that the vibration of the sensor can be rapidly attenuated. The structure can effectively improve the damping coefficient of the system, reduce the time of shock response, and eliminate the problem of signal adhesion [31]. The movable upper plate, middle plate, and fixed lower plate are all made of palladium silver (Pd-Ag) conductor material, and the ceramic dielectric layer is made of zirconia ceramic material. Due to the excellent characteristics of zero mechanical hysteretic, high elasticity, corrosion resistance, wear resistance, creep, small hysteretic, and high temperature robustness of the zirconia ceramic sheet, Hooke’s law is strictly followed until cracking. In addition, the strength of the material during compression is much higher than that during tension [32].

Generally speaking, the input acceleration range of a capacitive sensor structure with a circular plate is greater than that of a capacitive sensor structure with a square plate of the same size [31]. Therefore, the electrode plate and dielectric layer are designed to be circular [33]. The common operating principles of capacitive sensors include variable pole pitch, variable area, and a variable dielectric constant [34]. Considering the high impact force of projectile penetration, the capacitive sensor is designed as a variable-polar-pitch type using a high-strength, impact-resistant [35], and hard dielectric material, as shown in Figure 3.

In the three-pole plate capacitive sensor, the dielectric vibrates during the intrusion process and, at this moment, when the dielectric moves between the plates, damping is generated between the two plates. This damping includes the damping generated when the dielectric vibrates and the air damping caused by some air left in the gap during the manufacturing process. Damping is a destructive factor in maintaining vibrations. In many cases, measures are taken to reduce the damping so that vibration can be maintained at a minimum energy complement per cycle. However, in many other cases, damping is deliberately introduced into the system to reduce oscillations. A notable example is the micro-accelerometer. Damping is necessary, and proper damping should be considered from the design stage.

As shown in Figure 3b, since the lower pole plate is fixed, the upper pole plate and the middle pole plate can be equivalently regarded as masses *m*_1_ and *m*_2_. The stress–strain is transferred between each two pole plates through the dielectric. The electrode plate generates stress–strain through its own elastic deformation, and at the same time, there will be a damping effect. Similarly, the dielectric will have elastic deformation and damping effect in the process of transferring stress. Therefore, the elastic deformation and damping between the electrode plate and the dielectric are equivalent to the springer and the dampers, which constitute the dynamics model of the mass-spring-damping system. The kinetic equation of its kinetic model can be expressed as:

(1) $$\left[\begin{array}{cc} m_{1} & 0\\ 0 & m_{2} \end{array}\right]\left[\begin{array}{c} {\ddot{x}}_{1}\\ {\ddot{x}}_{2} \end{array}\right]+\left[\begin{array}{cc} c_{1}+c_{2} & -c_{2}\\ -c_{2} & c_{2} \end{array}\right]\left[\begin{array}{c} {\dot{x}}_{1}\\ {\dot{x}}_{2} \end{array}\right]+\left[\begin{array}{cc} k_{1}+k_{2} & -k_{2}\\ -k_{2} & k_{2} \end{array}\right]\left[\begin{array}{c} x_{1}\\ x_{2} \end{array}\right]=\left[\begin{array}{c} 0\\ F(t) \end{array}\right]$$

where *m*_1_, *m*_2_, *k*_1_, *k*_2_, *c*_1_, *c*_2_, *x*_1_ and *x*_2_ are the mass, stiffness, equivalent damping coefficient, and displacement of the middle and upper pole plates, respectively, and where *F*(*t*) = (*m*_1_ + *m*_2_) *a*_1_, *a*_1_ is the impact acceleration experienced by the sensor during penetration. The differential equation of Equation (1) can be transformed into a binary equation by Laplace variation as follows:

(2) $$\{\begin{array}{c} m_{1}s^{2}x_{1}(s)+(c_{1}+c_{2})sx_{1}(s)-c_{2}sx_{2}(s)+(k_{1}+k_{2})x_{1}(s)-k_{2}x_{2}(s)=0\\ m_{2}s^{2}x_{2}(s)-c_{2}sx_{1}(s)+c_{2}sx_{2}(s)-k_{2}x_{1}(s)+k_{2}x_{2}(s)=F(s) \end{array}$$

Then, *s* = *jω* is substituted into Equation (2) and the vibration amplitude of the upper and middle plates is obtained by complex operation and joint solution:

(3) $$x_{1}=\frac{cF(t)}{ab-c^{2}},x_{2}=\frac{bF(t)}{ab-c^{2}}$$

In the formula:

$$\begin{array}{l} a=k_{2}-m_{2}\omega^{2}+\omega c_{2}j;\;b=k_{1}+k_{2}-m_{1}\omega^{2}+\omega \left(c_{1}+c_{2}\right)j;\;\\ c=k_{2}+\omega c_{2}j;\;\omega =\sqrt{\frac{k_{2}}{m_{2}}}; \end{array}$$

The total capacitance of the sensor consists of two capacitance components formed by the three pole plates of the sensor:

(4) $$C=C_{1}+C_{2}$$

(5) $$C_{1}=\frac{\varepsilon_{0}\varepsilon_{r}A}{d_{0}-x_{1}},C_{2}=\frac{\varepsilon_{0}\varepsilon_{r}A}{d_{0}-x_{2}}$$

(6) $$\begin{array}{l} C_{1}=\frac{\varepsilon_{0}\varepsilon_{r}A}{d_{0}-x_{1}}=\frac{\varepsilon_{0}\varepsilon_{r}A}{d_{0}\left(1-\frac{x_{1}}{d_{0}}\right)}=\frac{\varepsilon_{0}\varepsilon_{r}A\left(1+\frac{x_{1}}{d_{0}}\right)}{d_{0}\left(1-\frac{x_{1}^{2}}{d_{0}^{2}}\right)}\\ C_{2}=\frac{\varepsilon_{0}\varepsilon_{r}A}{d_{0}-x_{2}}=\frac{\varepsilon_{0}\varepsilon_{r}A}{d_{0}\left(1-\frac{x_{2}}{d_{0}}\right)}=\frac{\varepsilon_{0}\varepsilon_{r}A\left(1+\frac{x_{2}}{d_{0}}\right)}{d_{0}\left(1-\frac{x_{2}^{2}}{d_{0}^{2}}\right)} \end{array}$$

Since the vibration amplitude is much smaller than the initial pole–plate spacing, Equation (6) can be reduced to the following equation:

(7) $$C_{1}=C_{0}-C_{0}\frac{x_{1}}{d_{0}},C_{2}=C_{0}-C_{0}\frac{x_{2}}{d_{0}}$$

Bringing Equation (3) into Equation (7) yields the relationship between acceleration and capacitance:

(8) $$C=C_{0}-C_{0}\left(\frac{(b+c)(m_{1}+m_{2})a_{1}}{d_{0}(ab-c^{2})}\right)$$

## 3. COMSOL Finite Element Simulation

### 3.1. COMSOL Mode Simulation

**Figure 4:**
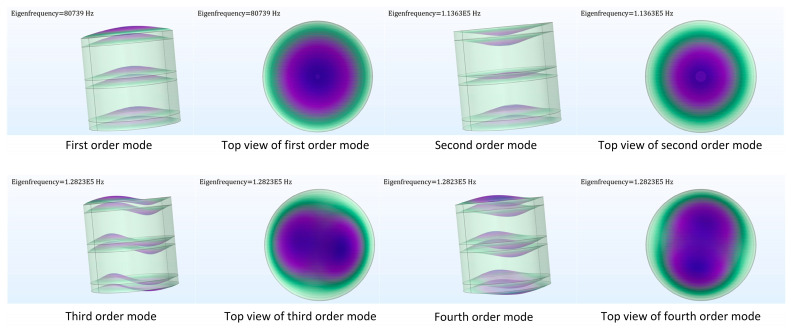
Fourth mode diagram of double-layer plate capacitive accelerometer.

The first four orders of modal simulation of the sensor structure were carried out using COMSOL software, and the simulation results are shown in Table 1. The simulation results show that the capacitor has an intrinsic frequency of 80.739 kHz in the direction of pulse acceleration in the first-order mode. The specific fourth-order modal diagram is shown in Figure 4

The frequency response cutoff frequency caused by the penetration impact is less than 20 kHz, and the natural frequency of the three-layer capacitor structure should be 3–5 times higher than the cutoff frequency of the target signal. This is to ensure the accuracy of the dynamic measurement. The first-order natural frequency of the three-layer plate capacitor sensor is 4 times the cutoff frequency of the target signal; therefore, the double-layer plate capacitor can meet the requirements of no measurement distortion.

### 3.2. Impact Simulation of Double-Layer Capacitive Accelerometer

In the process of projectile penetration, the triple electrode plate acceleration sensor will be subjected to a high impact, so the impact displacement simulation of the sensor plate is carried out to determine whether the impact displacement of the plate conforms to the theory of small deflection deformation.

**Figure 5:**
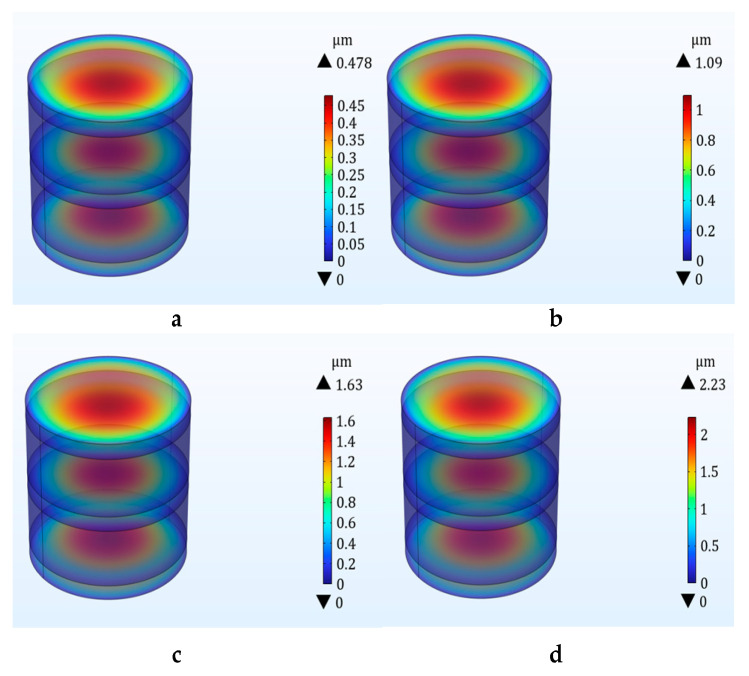
Shock displacement plots of double capacitance accelerometers under different shocks. (**a**) 40,000 g impact displacement map, (**b**) 100,000 g impact displacement map, (**c**) 150,000 g impact displacement map, (**d**) 200,000 g impact displacement map.

The response frequency during projectile penetration is about 20 kHz. The impact on the sensor during projectile penetration into the concrete slab is simulated by giving the capacitive accelerometer (a) 40,000 g/40 µs, (b) 100,000 g/40 µs, (c) 150,000 g/40 µs, and (d) 200,000 g/40 µs. This is shown in Figure 5. The maximum displacement at the top pole plate under the impact of 200,000 g/40 µs is 2.23 microns, which is consistent with the theory of small deflection deformation.

The sensor is affected by the penetrating stress wave at the tail of the projectile, and the impact acceleration of the sensor is about 10,000 g. Therefore, we conducted a 10,000 g impact analysis on the sensor and observed the maximum displacement and stress of the sensor. Through the simulation, we found that under the sinusoidal impact of 10,000 g/40 µs, the displacement of the sensor was 0.11 micron. The maximum stress was 0.136 MPa. It can be seen that the structure was relatively stable at a 10,000 g impact. We learn from Figure 2b that the impact of the entire projectile body upon penetration can reach 200,000 g.

Therefore, we carried out impact simulation on the sensor over the range, and the result was that the maximum displacement of the plate was 2.23 microns and the maximum stress was 68 MPa. It can be considered that the sensor has a stable structure under a 200,000 g impact and can still output linearly. The 200,000 g impact is the penetration impact size that the projectile as a whole can achieve. We use the maximum penetration impact to judge whether the sensor can stabilize output. Through simulation, we can see that the structure can withstand 200,000 g of impact without damage.

As shown in Figure 1, we conducted penetration simulation on the projectile, and the projectile carried out penetration motion at the initial velocity of 800 m/s. The acceleration of the projectile was with the process of projectile penetration into the cement plate and change. The specific value changes, as shown in Figure 2. Because the fuze is located at the tail of the projectile, when the peak impact of the penetration to the rear of the projectile is about 10,000–15,000 g, the natural frequency of the sensor is 80.739 kHz. The impact frequency caused by the impact of each layer of the projectile is about 20 kHz, that is, the time to penetrate each layer of the pole plate is 40 microseconds.

**Figure 6:**
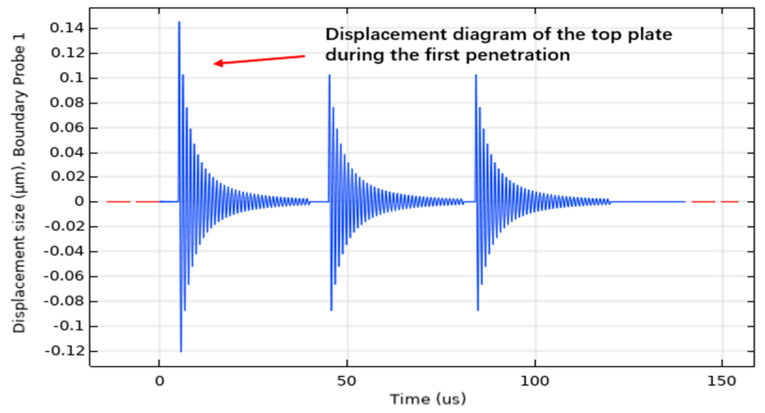
Sensor displacement curve.

Giving a peak acceleration of 10,000 g/120 µs to a three-layer electrode plate capacitive sensor, the equivalent displacement of the upper pole plate surface is shown in Figure 6. When the projectile penetrates the first layer of cement plate, the velocity is maximum and the displacement is at its maximum at this time; the cement plate will have a resistance to the projectile and the projectile will produce a reverse acceleration accordingly. The upper pole plate displacement gradually decreases as the acceleration decreases. In the second and third layers of the target, the peak displacement of the upper pole plate is smaller than that in the first layer of the target.

When the projectile penetrates the first layer of cement plate, the velocity is the maximum, at this time, the displacement is the maximum; when the acceleration decreases, the displacement of the upper pole plate gradually decreases.

## 4. Process Design of the Plate Capacitive Accelerometer

**Figure 7:**
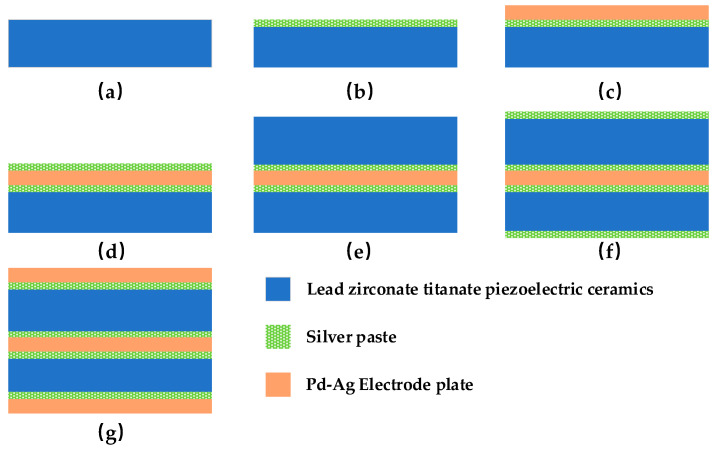
Schematic diagram of process steps. (**a**) PZT electrode plate. (**b**) Screen printing (**c**) Dry film lamination (**d**) Double-layer plate capacitor (**e**) Sintered two PZT plates (**f**) Top screen printing (**g**) Electrode plate welding.

The main process steps of the double-plate capacitor are shown in Figure 7 [36]. This is unlike [36], where we used lead zirconate titanate as a dielectric. The specific steps are as follows: (1) Firstly, two pieces of lead zirconate titanate piezoelectric ceramics (PZT) are machined to the designed dimensions by mechanical processing, as shown in Figure 7a. (2) Using the thick film process, the silver conductor paste was screen printed on the two ceramics by screen printing, as shown in Figure 7b. (3) Dry film lamination was then performed to form a photosensitive etched impedance layer to form a capacitive plate, as shown in Figure 7c. (4) The ceramic sheet was ground to form a complete double-layer plate capacitor structure, which was pre-sintered under an infrared light lamp for half an hour at room temperature to prevent the formation of structural bubbles. The plate molecules were then recrystallized in a tunnel furnace to improve the strength of the capacitor structure. Finally, it was cooled in a vacuum for 1.5 h. The results are shown in Figure 7d,e. (5) The upper and lower pole plates were brushed with silver conductor paste for dry film lamination, as shown in Figure 7f. (6) The prepared double-layer flat plate capacitor was taken out and the three flat plates were soldered together with leads to make a capacitive sensing element, as shown in Figure 7g.

## 5. Experiment and Analysis

### 5.1. Machette Hammer Impact Experiment

According to the simulation, the maximum impact on the projectile tail where the sensor is located during penetration is about 10,000 g, so a Machette’s hammer test was used for impact testing.

**Figure 8:**
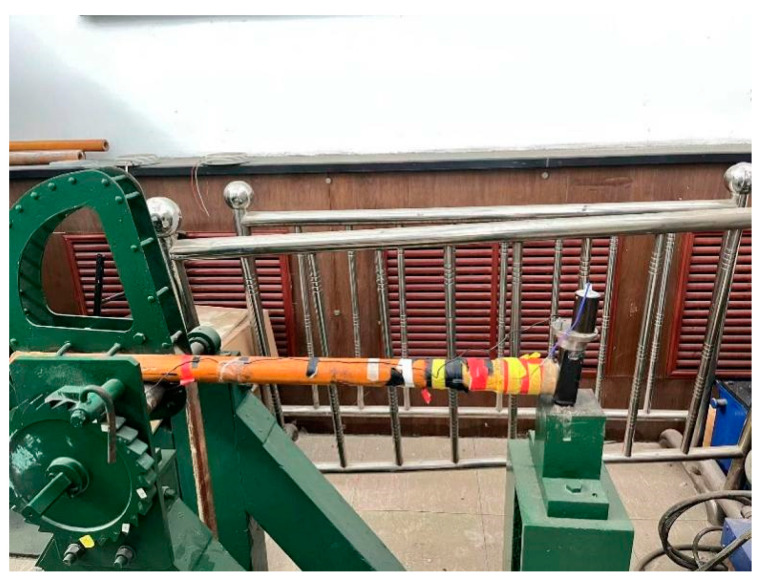
Machette’s hammer test equipment.

The test is an impact Machette shock test on the designed double-pole plate capacitor. The Machette hammer consists of four parts: the hammer head (fixture and sensor), the teeth, the weight, and the anvil. The test instrument is shown in Figure 8.

A certain acceleration is determined with a Machette hammer to simulate the force acting on the internal acceleration sensor when the projectile penetrates the concrete target. The effective measurement range of the sensor is 0–200,000 g. According to the simulation test, the target material is constructed with C30 concrete. When the initial penetration velocity is 800 m/s, the peak overload acceleration of the projectile body is usually in the range of 10,000–20,000 g. The maximum acceleration pulse width of the Machette hammer used was 120 µs, and the maximum impact acceleration was 50,000 g. Therefore, this experiment can simulate the penetration process of single-layer target.

After the sensor test platform was built, the Machette hammer was used to test the electrical performance of the sensor.

**Figure 9:**
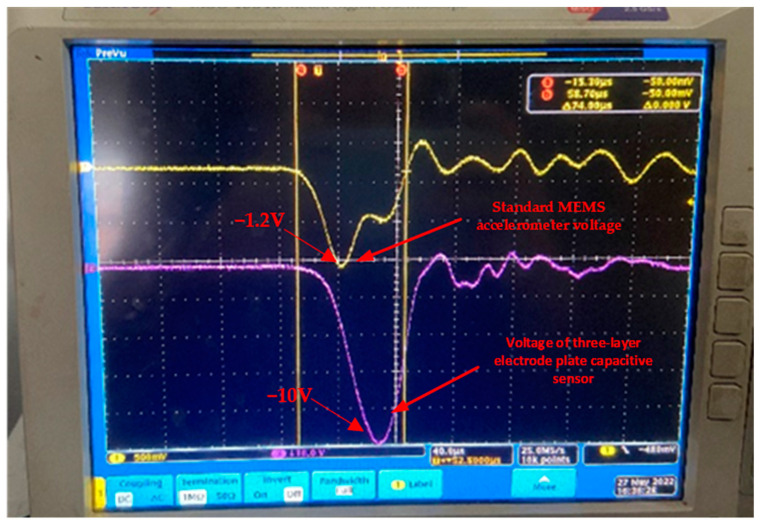
Voltage output diagram of the sensor.

Impact the sensor with a Machette hammer at 12,520 g. The output image of the sensor voltage is shown in Figure 9.

### 5.2. Data Analysis

From the purple waveform in Figure 8, it can be seen that the test experiment results in the signal change trend and simulation results remain consistent and the voltage signal output and the sensor changes are similar; in crossing a layer of cement board, the signal is also first sharply increased until it reaches a peak voltage of −10 V; the size of the peak voltage is also the sensor of the overload shock accurately perceived by the embodiment. After reaching the peak, the voltage falls back quickly until it reaches zero voltage. At this point, the plates squeeze each other and bounce back and the sensor voltage signal increases again; however, because the strength of the plate bounce back after the collision is small, and coupled with the material damping that exists from beginning to end and the continuous attenuation of the movement of the plates, the amplitude of the bounce back is small and the sensor bounce output voltage signal is also small, about a quarter of the peak voltage. The yellow signal in the figure is the output signal of the MEMS accelerometer, and the purple line represents the output response of the three-layer plate acceleration sensor. The MEMS accelerometer was compared with an Endevco accelerometer. With its high sensitivity and fast response time, the Endevco accelerometer is widely used in many fields, such as mechanical engineering, the automotive industry, aerospace, and medical equipment. It has great sensitivity and stability and is able to provide accurate measurement results over a wide range of temperatures and frequencies. At the same time, it is well designed to minimize noise interference and provide a clear acceleration signal. Additionally, the Endevco accelerometer has a range of 0–100,000 g and a sensitivity of 0.00001 V/g.

As can be seen in Figure 9, the response amplitude of the three-layer electrode plate capacitance sensor is large, the fallback is free of noise, and the response is superior to the output signals of the Endevco accelerometer used for the comparison.

## 6. Conclusions

In this paper, a dual-capacitance acceleration sensor based on three-layer flat plate is designed. Aiming at the problem of excessive clutter interference for the fuze overload signal in the process of target penetration, a theoretical model of the capacitive sensor is constructed from the perspective of a sensor by using the principle of a capacitive sensor. From the perspective of material, zirconia was selected as the dielectric material and a palladium–silver alloy as the plate material. The structure of double-layer plate capacitor was designed. The transient and modal simulation of the sensor is carried out by COMSOL simulation software, and the influence law of the structural parameters on the sensor performance is obtained and the appropriate parameter values are determined. Moreover, the three-dimensional overload finite element simulation analysis model of the full-size projectile penetrating the three-layer concrete target plate is established by LS-DYNA and the velocity acceleration of the projectile penetrating is analyzed. The manufacturing process of the double-plate capacitor is introduced. The sensor is processed and manufactured and the output characteristics of the sensor are tested using a Machette hammer. Finally, a capacitive acceleration sensor with stable output voltage and less clutter is obtained.

## Figures and Tables

**Table 1:** Fourth-mode frequency.

Modal Order	Vibration Frequency (kHz)
1	80.739
2	113.631
3	128.231
4	128.234

## Data Availability

Not applicable.
